# Identification of a rare p.G320R alpha-1-antitrypsin variant in emphysema and lung cancer patients

**DOI:** 10.1590/S1415-47572009005000100

**Published:** 2010-03-01

**Authors:** Mila Ljujic, Aleksandra Topic, Aleksandra Nikolic, Aleksandra Divac, Milan Grujic, Marija Mitic-Milikic, Dragica Radojkovic

**Affiliations:** 1 Institute of Molecular Genetics and Genetic Engineering, BelgradeSerbia; 2 Institute of Medical Biochemistry, Faculty of Pharmacy, Belgrade University, BelgradeSerbia; 3 Institute for Lung Disease and TB, University Clinical Center of Serbia, BelgradeSerbia

**Keywords:** alpha-1-antitrypsin, emphysema, lung cancer, P variant, specific trypsin inhibitory activity

## Abstract

The alpha-1-antitrypsin (A1AT) gene is highly polymorphic, with more than 100 genetic variants identified of which some can affect A1AT protein concentration and/or function and lead to pulmonary and/or liver disease. This study reports on the characterization of a p.G320R variant found in two patients, one with emphysema and the other with lung cancer. This variant results from a single base-pair substitution in exon 4 of the A1AT gene, and has been characterized as P by isoelectric focusing. Functional evaluation of the A1AT p.G320R variant was through comparing specific trypsin inhibitory activity in two patients with pulmonary disorders, carriers of the p.G320R variant, and 19 healthy individuals, carriers of normal A1AT M variants. Results showed that specific trypsin inhibitory activity was lower in both emphysema (2.45 mU/g) and lung cancer (2.07 mU/g) patients than in carriers of the normal variants (range 2.51-3.71 mU/g). This rare A1AT variant is associated with reduced functional activity of A1AT protein. Considering that it was found in patients with severe pulmonary disorders, this variant could be of clinical significance.

Alpha-1-antitrypsin (A1AT) is the most abundant circulating plasma serine proteinase inhibitor and functions as a major inhibitor of neutrophil elastase in the lower respiratory tract. The gene coding for A1AT spans over 12.2 kb on chromosome 14, and is comprised of seven exons and six introns (Crystal, 1990). The two parental A1AT genes are codominantly expressed and contribute to A1AT serum concentrations. Human A1AT is a 52 kDa, 394 amino-acids-long acute phase glycoprotein composed of nine α helices (A → I), three β-sheets and a mobile reactive loop, and is mainly synthesized in the liver (Lomas and Mahadeva, 2002; Lomas and Parfey, 2004). The core of the reactive center within the loop is represented by two amino acid residues, Met-358 and Ser-359. The enzyme reacts as bait or a mouse trap for its target proteinases. The complex between A1AT and its target is recognized by hepatic receptors and cleared from circulation.

The A1AT gene is highly polymorphic, with more than 100 genetic variants identified, so far (Luisetti and Seersholm, 2004). A1AT protein variants are categorized as normal, deficient, null or dysfunctional (American Thoracic Society/European Respiratory Society Statement, 2003). Deficient variants are associated with reduced A1AT serum concentrations, undetectable in the case of null variants. Dysfunctional variants are associated with normal A1AT serum concentrations, but have altered activity against target proteinases. The most common mutated A1AT variant is Z, which is categorized as both deficient and dysfunctional (Brantly *et al.*, 1996). Adequate concentrations and normal functioning of A1AT protein provide a critical defense against proteolytic stress. Abnormalities in both concentration and function of A1AT result in predisposition to emphysema and in the development of cirrhosis (Carrell and Lomas, 2002). The association of A1AT genetic variants with several other clinical conditions, such as asthma, chronic obstructive pulmonary disease, lung cancer, pancreatitis and liver cancer, has also been reported (Lomas and Parfrey, 2004; Yang *et al.*, 2008).

This study reports on the functional evaluation of an A1AT p.G320R variant found in two patients with severe pulmonary disorders, emphysema and lung cancer. This rare variant was previously described in one of 50 025 patients analyzed in a retrospective study in the USA (Bornhorst *et al.*, 2007). In our study, the functional evaluation of the p.G320R variant was carried out by determining specific trypsin inhibitory activity (STIA) in both patients and 19 healthy individuals, carriers of normal A1AT alleles M1, M2 and M3 (M1, M2, M3, M1M2 and M1M3). The protocol was approved by local research ethics committees, and informed consent was obtained from each participant in the study.

The patient with emphysema was a 31-year-old male, a non-smoker, with a past history of repeated respiratory infections in childhood. Chest X-ray and chest computed tomography (CT) scanning revealed hyper-inflated lung fields with attenuation of peripheral vasculature, flattened diaphragms and increased retrosternal clear space, with patchy opacification in the upper part of the lungs. Auscultation revealed weakened breathing-sounds with prolonged expiration. Pulmonary function tests revealed a severe degree of ventilatory impairment (FVC of 81% predicted, postbronchodilatator FEV_1_ of 29% predicted, FEV_1_/FVC of 30% predicted). Arterial blood gas results were normal as were diffusing capacity and diffusing coefficient of the lung. Perfusion scintigraphy (scan) showed bilateral zones of hypo-perfusion and defects. Sputum examination was positive for bacterial infection with *Pseudomonas aeruginosa*. There was no evidence of liver disease.

The patient with lung cancer was a 73 year-old male, smoker (240 pack years), with a positive family history of chronic obstructive pulmonary disease and lung cancer, and with clinical signs of Superior vena cava syndrome. Microcellular lung carcinoma was diagnosed by bronchoscopic biopsy and biopsy of the neck lymph node. Chest X-ray showed an enlargement of the upper mediastinum. CT scanning confirmed a large (110 x 65 mm) mass causing compression of the vascular structure on the right lung, with the enlargement of supraclavicular lymph nodes on the right side. Pulmonary function tests revealed a severe ventilatory impairment (FVC of 59% predicted, FEV_1_ of 36% predicted, FEV_1_/FVC of 46% predicted) and hypoxemia (PaO_2_ 8.2 kPa) without hypercapnia in arterial blood. Biochemical analyses, ultrasound and CT scanning indicated normal liver morphology and function.

Isoelectric focusing (IEF) of serum proteins, performed on the pH range 4.2-4.9, with the 2117 Multiphor system (GE Healthcare) revealed the M1P phenotype in both patients (Figure 1A).

The whole A1AT gene was analyzed by direct DNA sequencing, using the ABI Prism BigDye Terminator Kit (Applied Biosystems). Sequence analysis with Sequence Analysis Software (Applied Biosystems) revealed the presence of a p.G320R (c.1030G > A) variant in both patients (GenBank accession number EF683685) (Figure 1B). This variant results from a single base-pair substitution of guanine by adenine in exon 4 of the A1AT gene (Gly-320[GGG] → Arg-320[AGG]). In both patients, p.G320R occured on the common normal M1Val213 genetic background.

Three-dimensional (3D) models of the A1AT wild-type and p.G320R variant, generated using Swissmodel, were analyzed by Matras Protein 3D Structure Comparison (Guex and Peitsch, 1997; Kawabata, 2003). No structural differences between wild-type protein and mutated proteins were observed.

In order to evaluate whether p.G320R variant has altered activity in comparison to the wild type protein, specific trypsin inhibitory activity (STIA) values were determined in both carriers of A1AT p.G320R variant, as well as in 19 healthy individuals, carriers of normal A1AT alleles M1, M2 and M3 (M1, M2, M3, M1M2 and M1M3). Serum A1AT concentration was measured on NOR-Partigen plates (Dade Behring). Serum trypsin inhibitory capacity (TIC) was measured with N-benzoyl-dl-arginine-p-nitroaniline (BAPNA, Sigma) as substrate by the method of Dietz and coworkers which was adapted for IL 600 Clinical Chemistry Analyzer (Instrumentation Laboratory) (Dietz, 1976). All TIC measurements were performed in triplicate. To assess the functionality of A1AT molecules and to avoid the influence of A1AT concentration, the STIA was calculated as the ratio between measured TIC values and serum A1AT concentrations. Although according to the concentration, the p.G320R variant appears not to be deficient, its STIA values were reduced in both emphysema (2.45 mU/g) and lung cancer (2.07 mU/g) patients in comparison with those obtained in healthy individuals (3.11 ± 0.30 mU/g) (Table 1).

Our study on functional evaluation of A1AT p.G320R variant by determination of specific trypsin inhibitory activity demonstrated its reduced activity in comparison to normal A1AT M variants. Although molecular modeling revealed no 3D differences between wild-type and mutated protein, specific trypsin inhibitory activity of p.G320R variant was significantly lower in comparison to normal M variants. In the p.G320R variant, the mutated amino acid is located outside the reactive center loop of the protease inhibitor, in a hinge region between the αI and the β5A sheet. The β5A sheet, together with β3A and neighboring sites, is a part of the shutter region in the serpin molecule, the latter being important in controlling and modulating conformational changes during sheet opening and insertion of the conserved hinge of the reactive center loop (Whisstock *et al.*, 2000; Irving *et al.*, 2000). Numerous mutants identified in the shutter region of the serpin molecule lead to serpin dysfunction and subsequent serpinopathies (Stein and Carrell, 1995). In the case of the p.G320R variant, there is the possibility that a more voluminous, positively charged amino acid (Arg) replacing a small, nonpolar one (Gly) might influence conformation and thus affect A1AT functional activity.

According to its migration pattern on IEF gels, the p.G320R variant phenotype has been characterized as P and recently denoted as P_salt lake_ (Bornhorst *et al.*, 2007), but to our best knowledge, nothing has been published regarding further characterization of this rare variant. Several different genetic variants have been shown to result in the “P” phenotype, but they are indistinguishable by IEF, and therefore have not been well studied. The group of “P” variants includes those associated with reduced (P_lowell_, P_duarte_) and normal (P_st albans_, P_budapest_) serum A1AT concentrations, but little is known on inhibitory activity associated with different “P” variants. One of the best studied “P” variants, P_lowell_, is associated with reduced serum A1AT concentrations and near normal inhibitory activity. It was also discovered in a case of severe A1AT deficiency (Cook *et al.*, 1995). In our study, the p.G320R variant (P_salt lake_) was found in two patients with severe pulmonary disorders, and was associated with reduced inhibitory activity.

Findings of this study, along with other studies reporting on variability of A1AT “P” phenotype, emphasize the need for caution in clinical interpretation of results obtained by IEF.

In summary, this study demonstrated impaired activity of serum A1AT in carriers of p.G320R variant, which appears not to be deficient. Considering that it was found in patients with severe pulmonary disorders, this variant might be of clinical significance. These findings should be confirmed by analyzing the specific trypsin inhibitory capacity of recombinant A1AT p.G320R variant in order to completely eliminate the influence of different physiological factors that might interfere with protein activity.

**Figure 1:**
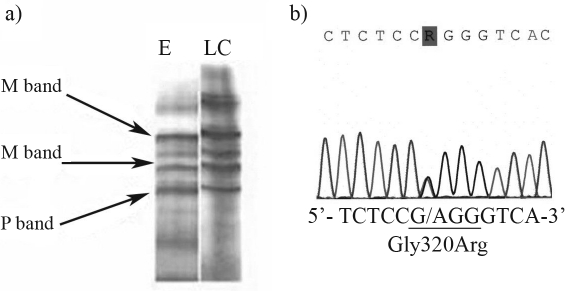
A. Phenotyping of A1AT in serum samples of patients with emphysema (E) and lung cancer (LC) by isoelectric focusing. Major M and P bands are denoted by arrows. B. DNA sequence of p.G320R variant.

## Figures and Tables

**Table 1:** Concentrations, trypsin inhibitory capacity (TIC) and specific inhibitory activity (STIA) of A1AT in healthy individuals with MM variants (n = 19) and in patients-carriers of p.G320R variant.

A1AT genotype	Concentration of A1AT (g/L)	TIC (mU)	STIA (mU/g)
MM	1.53 ± 0.138	4.73 ± 0.507	3.11 ± 0.354
M1p.G320R-(emphysema patient)	0.97	2.37	2.45
M1p.G320R (lung cancer patient)	2.17	4.49	2.07
